# Secondary acute myeloid leukemia after successful treatment for osteosarcoma

**DOI:** 10.4103/0971-5851.68852

**Published:** 2010

**Authors:** Rakesh Mittal, N. V. Ramaswamy, R. Pandita, S. Al Bahar, N. Khalifa, S. Omar

**Affiliations:** *Unit of Pediatric Oncology, Department of Medical Oncology, Kuwait*; 1 *Hematology, Kuwait Cancer Control Centre, Kuwait*

**Keywords:** *Acute myeloid leukemia*, *chemotherapy*, *osteosarcoma*, *secondary malignancy*

## Abstract

Secondary acute myeloid leukemia (sAML) is a rare complication following chemotherapy for osteogenic sarcoma. However, the exact offending drug is difficult to prove as there is no consistent data. It usually develops 2 years after completion of therapy. We report a case of sAML that developed within 8 months of completing the treatment. The patient was treated with cisplatin, doxorubicin and high-dose methotreaxate followed by surgery (amputation). Eight months after completion of therapy, while on follow-up, he presented with leukocytosis and thrombocytopenia and confirmed to have AML.

## INTRODUCTION

Secondary acute myeloid leukemia (sAML) is an uncommon serious second malignant neoplasm (SMN) following chemotherapy for various cancers. The most common offending drugs are alkylating agents and topoisomerase II inhibitors. Among the topoisomerase II inhibitors, epipodophyllotoxins are the most common drugs responsible for sAML.[1–5]

Osteosarcoma (OS) is the most common malignant bone tumor that affects adolescents and young adults. It is treated by preoperative chemotherapy, surgery and postoperative chemotherapy. Various SMNs have been reported in OS patients.[6–9] sAML as a rare complication of OS has also been reported in the literature.[10–14] We report a case of sAML in an 8-year-old child within 8 months of completing treatment for OS.

## CASE REPORT

This 8-year-old boy was diagnosed with nonmetastatic osteosarcoma of the left lower femur. He was treated with neoadjuvant chemotherapy with PAM protocol (cisplatin, doxorubicin, high-dose methotrexate). A reduction of tumor size was noted after presurgery chemotherapy. However, as the tumor was radiologically close to the posterior vessels, he was not considered for limb conservation surgery and underwent transfemoral amputation. Histopathology showed good response to preoperative chemotherapy (more than 90% necrosis). After amputation, the patient was continued on postoperative chemotherapy. He received total cumulative doses of cisplatin (720 mg/m^2^), doxorubicin (450 mg/m^2^) and high-dose methotrexate (144 g/m^2^). After completion of the therapy, he was on regular follow-up every 2 months with blood tests and radiological examination of the local area and chest.

**Figure 1:**
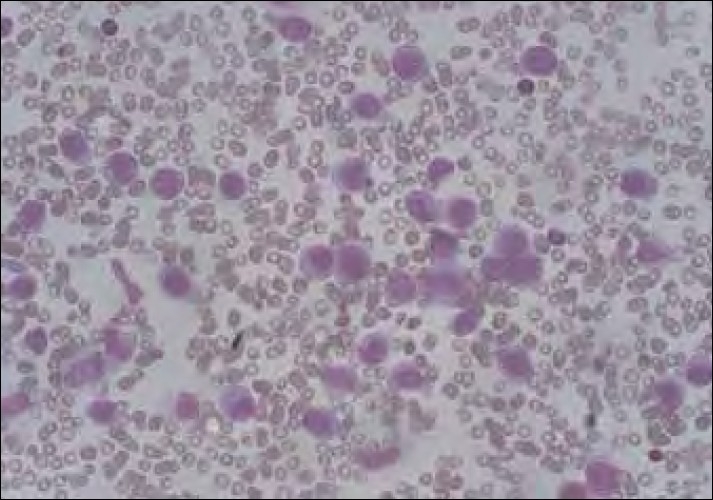
Bone marrow morphology showing acute myeloid leukemia

Within 8 months of treatment completion, he presented with multiple purpuric spots over the neck and arms. The complete blood count showed a white blood cell count of 45.3×10/l, Hb of 13.2 g/l and platelets 6×10/l, with the peripheral smear showing 41% blasts. Bone marrow examination revealed hypercellularity with marked suppression of normal hematopoiesis and extensive infiltration by blasts (71%). Blasts were large, with moderate to abundant lightly basophilic cytoplasm. Some cells were vacuolated while others contained fine azurophilic granules. Nuclei were round and folded containing, one to three nucleoli [Figure 1]. On cytochemistry, the blasts were nonspecific estrase+++ (fluoride sensitive), Sudan black++, peroxidase+ and periodic acid Schiff stain diffuse weak+. Immunophenotyping analysis of gated cells showed that blasts expressed CD13, CD33, CD14, HLA-DR, CD68 and myeloperoxidase. Diagnosis of acute myelomonocytic leukemia was confirmed on the basis of morphology, cytochemistry and immunophenotyping (FAB-M4). On karyotyping, 30 metaphases were analyzed, of which 29 showed 46XY and the remaining one showed -5. Applying the 5q31/5p15 probe on bone marrow interphase cells, two chromosomes 5 were detected in 95% of the cells. Because the monosomy of chromosome 5 was detected only in one of the 30 examined metaphases, it was not of clonal origin. Before initiation of the treatment, the patient had severe pulmonary hemorrhage, to which he succumbed.

## DISCUSSION

Development of SMNs in osteosarcoma patients is not uncommon. The 10-year cumulative incidence of SMN in OS patients reported varies from 2 + 1%,[6] to 4.6%.[8] This is higher than the expected rate for benign bone tumors,[8] but is less than the reported incidence for Hodgkin’s disease (9.7%)[15] and retinoblastoma patients (30%).[16] Majority of the SMNs in OS reported in the literature are solid tumors of various organs or tissues.[6–9] Presence of p53 mutation (Li Frumani syndrome) in OS patients increases the incidence of nontherapy-related synchronous or metachronous SMNs.[17] Our patient had no family history of malignancy. The occurrence of sAML in OS is a rare complication. Very few cases have been reported either as part of large series of SMNs in OS[6–9] or as individual case reports.[10–13] The median time to develop SMNs after OS varies from 5.5 years to 7.6 years.[6–8] The sAML developing after alkylating agents will have preceding myelodysplasia, long latency period and monosomy 5 or 7 with AML (FAB type M1 or M2). sAML after topoisomerase II inhibitors have shorter latent time, FAB M4-M5 type and have translocation involving the MLL gene at chromosome band 11q23. The common topoisomerase II inhibitors are epipodophyllotoxins (etoposide, tenoposide) and anthracyclines (adriamycin, mitoxantrone). The reported minimum time to develop sAML was generally more than 1 year. However, Escudero *et al*,[13] reported that one of their case developed sAML after 7 months while our case developed sAML after 8 months. This is less than the time period for sAML after therapy with epipodophyllotoxins. A majority of the reported cases of sAML after topoisomerase II inhibitors are due to etoposide.[3] The incidence of sAML in patients who received anthracyclins for the treatment of their primary tumors is not well known. In the series from Bacci *et al*.,[8] of the 35 patients who received only anthracyclines, none developed sAML. The common drugs used for chemotherapy in OS are adriamycin, cisplatin and high-dose methotrexate, but ifosfamide, vincristine, bleomycin and carboplatin are also used. These chemotherapeutic drugs are not attributed to sAML commonly. Cisplatin has been reported for sAML after germ cell tumors in young patients,[18] but not after OS treatment. It has been postulated that sAML in OS patients could be a synergistic effect of anthracyclins and cisplatin, which could be the likely cause in our case.[13]

Pyatt *et al*,[2] found no consistent relation between age at which chemotherapy was given and development of sAML, and concluded that younger age was not a risk factor for the development of sAML. The time interval between completion of chemotherapy for the primary tumor and development of sAML depends on the type of chemotherapy used. Escudero *et al*,[13] reported a case of sAML after treatment for OS, who developed myelodysplastic syndrome 9 months after completion of chemotherapy for OS and evolved into sAML after 14 months. He also had deletion of long arm of chromosome 7. Because that patient had a short latency period and had only one dose of ifosphamide, the authors could not confirm the offending drug as alkylating agent.

Kawai *et al*.,[12] while presenting two cases of sAML after OS treatment, compiled a series of 16 cases of acute leukemia following treatment for primary OS. In that series, there were only 10 confirmed cases of sAML. Three had prior myelodysplastic syndrome, of which two developed sAML. The most consistent cytogenetic abnormality was t(8q;21q), which was seen in five patients. Our patient did not have any clonal cytogenetic abnormality. The prognosis of the patients with sAML is generally considered poor compared to *de novo* AML.[19] Review of SEER data showed 5-year survival in sAML as 23.7%.[1] However, some authors have refuted this fact and have reported that survival could be equal to *de novo* AML, depending on the karyotyping abnormality.[20]

In conclusion, sAML is a rare complication after chemotherapy for OS. In a majority of the cases, it is related to the chemotherapeutic drugs used for the treatment of OS. However, the exact offending drug is difficult to prove as there is no consistent data. In view of the early onset of sAML reported in the literature, as in our case, patients need to be followed-up not only for the recurrence of OS but also for sAML.
